# Health Professionals' Expectations Versus Experiences of Internet-Based Telemonitoring: Survey Among Heart Failure Clinics

**DOI:** 10.2196/jmir.2161

**Published:** 2013-01-10

**Authors:** Arjen E de Vries, Martje H.L van der Wal, Maurice M.W Nieuwenhuis, Richard M de Jong, Rene B van Dijk, Tiny Jaarsma, Hans L Hillege

**Affiliations:** ^1^University Medical Center GroningenCardiologyGroningenNetherlands; ^2^Martini HospitalCardiologyGroningenNetherlands; ^3^Linkoping UniversityDepartment of Social and Welfare StudiesNorrkopingSweden

**Keywords:** Telemonitoring, Telemedicine, Remote monitoring, Internet, Heart failure, Heart failure management, Disease management,

## Abstract

**Background:**

Although telemonitoring is increasingly used in heart failure care, data on expectations, experiences, and organizational implications concerning telemonitoring are rarely addressed, and the optimal profile of patients who can benefit from telemonitoring has yet to be defined.

**Objective:**

To assess the actual status of use of telemonitoring and to describe the expectations, experiences, and organizational aspects involved in working with telemonitoring in heart failure in the Netherlands.

**Methods:**

In collaboration with the Netherlands Organization for Applied Scientific Research (TNO), a 19-item survey was sent to all outpatient heart failure clinics in the Netherlands, addressed to cardiologists and heart failure nurses working in the clinics.

**Results:**

Of the 109 heart failure clinics who received a survey, 86 clinics responded (79%). In total, 31 out of 86 (36%) heart failure clinics were using telemonitoring and 12 heart failure clinics (14%) planned to use telemonitoring within one year. The number of heart failure patients receiving telemonitoring generally varied between 10 and 50; although in two clinics more than 75 patients used telemonitoring. The main goals for using telemonitoring are “monitoring physical condition”, “monitoring signs of deterioration” (n=39, 91%), “monitoring treatment” (n=32, 74%), “adjusting medication” (n=24, 56%), and “educating patients” (n=33, 77%). Most patients using telemonitoring were in the New York Heart Association (NYHA) functional classes II (n=19, 61%) and III (n=27, 87%) and were offered the use of the telemonitoring system “as long as needed” or without a time limit. However, the expectations of the use of telemonitoring were not met after implementation. Eight of the 11 items about expectations versus experiences were significantly decreased (*P*<.001). Health care professionals experienced the most changes related to the use of telemonitoring in their work, in particular with respect to “keeping up with current development” (before 7.2, after 6.8, *P*=.15), “being innovative” (before 7.0, after 6.1, *P*=.003), and “better guideline adherence” (before 6.3, after 5.3, *P*=.005). Strikingly, 20 out of 31 heart failure clinics stated that they were considering using a different telemonitoring system than the system used at the time.

**Conclusions:**

One third of all heart failure clinics surveyed were using telemonitoring as part of their care without any transparent, predefined criteria of user requirements. Prior expectations of telemonitoring were not reflected in actual experiences, possibly leading to disappointment.

## Introduction

Telemonitoring in heart failure care is used to monitor patients’ symptoms at home and to guide patients in taking action in case of deterioration. Telemonitoring is considered a promising new intervention for heart failure patients, and a study on the use, perceptions, and experiences has been published recently [1,2]. However, current evidence regarding the effectiveness of telemonitoring in the care of heart failure patients is conflicting [3]. There are many definitions used for telemonitoring, but the core principle does not generally differ. A commonly used international definition is “the remote monitoring of patients, including the use of audio, video, and other telecommunications and electronic information processing technologies to monitor patient status at a distance” [4]. In the Netherlands, the most used definition is that telemonitoring includes the measurement, monitoring, collecting, and transfer of clinical data concerning the health status of a patient in his or her home environment, using information and communication technology. Initial studies showed that remote monitoring of heart failure patients reduced hospitalization and mortality rates [5-8]. However, recent studies performed on a larger scale did not confirm these findings [9,10]. Questions remain regarding the optimal patient profile for using telemonitoring, the technical aspects of the telemonitoring systems, the intensity and frequency of providing data, and the cost-effectiveness of the various telemonitoring systems used [11,12]. Furthermore, expectations and consequences of telemonitoring for the organization of care, logistic processes, and the work of health care providers are rarely studied, and thus unclear. However, these aspects of telemonitoring are vital for the consideration and acceptance of these systems in future practice [13].

Despite the inconclusive evidence for the use of telemonitoring in heart failure, telemonitoring is considered to be a promising development, [7] and there are increasing efforts to introduce telemonitoring in outpatient heart failure clinics. In some countries, including the Netherlands, health care insurance companies reimburse telemonitoring for heart failure patients. The present study was designed to assess the perspectives and expectations for both heart failure nurses and cardiologists working in a heart failure team with telemonitoring.

To this end, the following research questions were posed: 1) What are the perceptions and expectations of cardiologists and heart failure nurses with respect to the implementation of telemonitoring in heart failure patients? and 2) What are their experiences with the implementation of telemonitoring? In this study, we did not focus on possible differences between heart failure nurses and cardiologist in their perceptions of working with telemonitoring.

## Methods

### Participants

Participants in the study consisted of cardiologists and heart failure nurses working in heart failure outpatient clinics in the Netherlands. Out of all 118 Dutch heart failure clinics, 109 clinics received a questionnaire in March 2011, addressed to the cardiologists and heart failure nurses working in the heart failure outpatient clinic. Nine heart failure clinics were excluded and did not receive a questionnaire due to their participation in the IN TOUCH study, a study evaluating the added value of information and communication technology-guided disease management combined with telemonitoring for heart failure patients [14]. Participants were requested to return the questionnaire within 12 weeks. We sent out two reminders.

### Instrument

In collaboration with the Netherlands Organization for Applied Scientific Research (TNO), a 19-item questionnaire on telemonitoring was specifically developed for this study, based on the two research questions. For this questionnaire we defined telemonitoring as: “The remote, Internet-based monitoring and mentoring of heart failure patients on weight, blood pressure, heart rate, and signs and symptoms that disclose the actual condition of the heart failure patient. The devices are used by the patients in their own home environment and the generated data are transferred by the Internet”. The use of telemonitoring by means of telephone, telephone support, telephone follow-up, or by means of implantable devices was not included in this study because our focus was to investigate expectations and experiences of using telemonitoring devices that required an active user interaction (eg, direct handling of deviated values, generated alerts, and complaints). The technology and handling for users between implanted devices and external devices, such as weight scales and/or blood pressure measurements, are essentially different. Based on the research questions, items for the questionnaire were developed with the input of 10 cardiologists and 10 heart failure nurses, resulting in a questionnaire consisting of 3 domains: 1) availability of telemonitoring, 2) experiences with telemonitoring, and 3) organization of telemonitoring. The questionnaire consisted of both multiple choice and “agree/disagree” questions. For data regarding the motivation for and importance of using telemonitoring, as well as the experiences with using telemonitoring, we asked respondents to rate 11 items on a 10-point scale. On this scale, 0 counted as “not important”’ and 10 as “very important”.

These 11 items were based on practical considerations related to the start-up of telemonitoring. Aside from addressing the practical considerations of health care workers in our study, these same 11 items are frequently used by sales representatives to convince future users of the added value of working with telemonitoring. The 11 different items could be combined into 3 groups: 1) direct patient care (better self-management, improving quality of care, and reduction of (re) admission); 2) telemonitoring system–related aspects (current development, innovation, and better guideline adherence); and 3) organizational aspects (treating more patients, fulfilling hospital policy, reducing workload, lowering heart failure related costs, and fulfilling health care insurance policy).

### Validation Process of the Questionnaire

To test the questionnaire, a group of 30 pilot responders, representing the future research population, completed the questionnaire. Internal consistency (Cronbach alpha) of the questionnaire in the current sample was .85. This parameter measures the reliability of the scale. A set of questionnaire items with a reliability of .70 or higher is considered acceptable. Face validity (10 cardiologists, 10 heart failure nurses) was assessed by analyzing the feedback received on the total questionnaire.

### Statistical Analysis

Descriptive statistics were used to present the data. For some parts of the analysis, we subdivided the respondents into current telemonitoring users (n=31) and intended telemonitoring users (n=12), because some research questions are related to actual experiences of working with telemonitoring and other are more exploratory (eg, which patients do you think are suitable for applying telemonitoring?). Paired samples *t* tests were used to examine possible differences between expectations of and experiences with using telemonitoring. Analyses were performed using PASW, version 18.0 for Windows.

## Results

### Basic Characteristics of the Study Population

Of the 109 heart failure clinics who received a survey, 86 clinics responded (79%). Their responses were included in the analysis. Respondents had a mean age of 48 ± 8 years, and 68% were female. The mean years of work experience in the current position was 14 ± 9 years, and the respondents worked with heart failure patients for an average of 19 ± 10 hours a week. Of the 86 responding clinics, 31 reported using telemonitoring in their current patient care (36 %), and 12 clinics (14%) planned to use telemonitoring within one year. Further analysis was therefore restricted to the clinics that actually used telemonitoring and those that planned to use telemonitoring within one year (total n=43).

### Availability of Telemonitoring

The three systems most frequently used for telemonitoring were commercially available systems (Motiva, Health Buddy, and IPT Telemedicine [15-17]), and one clinic had developed its own telemonitoring system. The systems used in this study are generally similar to each other based on functionality. They transfer measurements generated at home and answers to questions to a health care environment via the Internet. The Health Buddy system differs, however, because it transfers the data directly to the health care provider instead of a data center. This means that the heart failure nurses are directly responsible for the handling of data and measurements. However, the consequence of directly receiving data and measurements is the need for a 24/7 shift of health care providers.

The feedback from the health care provider to the patient in all three systems is given by telephone. For the specific characteristics of the commercially available systems used in this study [18], see Table 1.

**Table 1:** Characteristics of the commercial available telemonitoring systems used in this study.

		Motiva	Health Buddy	IPT-Telemedicine
**Monitoring**				
	Blood pressure	yes	yes	yes
	Weight	yes	yes	yes
	Heart frequency	yes	yes	yes
	Electrocardiography	no	yes	yes
**Questions**				
	Symptoms	yes	yes	yes
	Knowledge about heart failure	yes	yes	yes
	Change of behavior	yes	yes	yes
**Informing patient about…**				
	Symptoms	yes	yes	yes
	Knowledge about heart failure	yes	yes	yes
	Change of behavior	yes	yes	yes
**Communication**				
	Datacenter	yes	yes	yes
	Medical service center	yes	no	yes
	Direct feedback, true application to patient	yes, through television	yes	yes
	Direct feedback from health care provider to patient	yes, by phone	yes, by phone	yes, by phone
	Continue feedback to health care provider	yes, through software on desktop	yes, through software on desktop	yes, through portal
	Alerts in case of deviation from predefined measurements	yes, through software on desktop	yes, risk profiles (low-middle-high)	yes, through portal
**Patient requirements**				
	Ability to read	yes	yes	yes
	Active input	yes	yes	yes
	Cognitive functional	yes	yes	yes
	Manual	extensive	simple	simple
	Television	yes	no	no

The 12 clinics that intended to use telemonitoring within a year mostly reported (42%, n=5) that they planned to use the Motiva system (Table 2). The number of patients using telemonitoring in a clinic varied between 10 and 50, but in two clinics more than 75 patients used telemonitoring.

**Table 2:** Availability and use of telemonitoring (TM) system by actual users (n=31) and planned users (n=12).

TM systems	Actually used system (n=31 clinics)	System of choice in case of a new decision (n=31 clinics)	No current user but expecting to make a choice within 1 year (n=12 clinics)
Health Buddy	7 (28%)	2 (8%)	–
Motiva	14 (46%)	4 (12%)	5 (42%)
IPT Telemedicine	6 (15%)	2 (6%)	–
Other systems	4 (11%)	3 (10%)	2 (16%)
No choice yet	–	4 (12%)	2 (16%)
Unsure	–	16 (52%)	3 (26%)

The following main goals for implementing telemonitoring were reported: “monitoring physical condition”, “monitoring signs of deterioration” (91%, n=39), “monitoring treatment” (74%, n=32), “adjusting medication” (56%, n=24), and “educating patients” (77%, n=33) (see Table 3). Beside these goals, most clinics also used this as a practical reason to start telemonitoring.

**Table 3:** General descriptive data of heart failure centers using (n=31) and planning to use (n=12) telemonitoring (TM).

Question (n)	Response option	Response n (%)
**Number of patients in TM care** (n=31 clinics)		
	None	2 (6%)
	0-10	5 (16%)
	10-20	8 (26%)
	20-50	11 (35%)
	50-75	3 (11%)
	>75	2 (6%)
**Main goal of using telemonitoring** (n=43 clinics, more than one answer possible)		
	Monitoring physical conditioning, signs of deterioration	39 (91%)
	Monitoring and adjustment of treatment	32 (74%)
	Titration of medication	24 (56%)
	Patient education	33 (77%)
	Other goals	3 (7%)
**Duration of applying telemonitoring in patient care** (n=31 clinics)		
	Between 3 and 6 months	6 (19%)
	Between 6 and 12 months	6 (19%)
	No limit	9 (30%)
	As long as necessary	10 (32%)

### Experience With Telemonitoring

#### Patient Profile

The criteria for using telemonitoring for a specific patient were reported to be based on “needing education” (68 %, n=29), “increasing self management” (63%, n=27), “having complaints of heart failure symptoms” (60%, n=26), and “being (re) admitted due to heart failure” (60%, n=26). See Table 4.

**Table 4:** Criteria for applying telemonitoring in heart failure (HF) patients.

Criteria for applying telemonitoring	n=43 clinics
Education	29 (68%)
Patient management	27 (63%)
Heart failure re-admission	26 (60%)
Complaints heart failure symptoms	26 (60%)
Based on actual NYHA class	13 (30%)
Medication status	8 (19%)
Different	2 (4%)

Respondents from 8 clinics reported that the current use or amount of medication were reasons for using telemonitoring. The majority of respondents (85%, n=36) stated that the New York Heart Association (NYHA) functional class was not a reason to start telemonitoring (see Table 5).

**Table 5:** NYHA class in telemonitoring (NYHA: New York Heart Association classification for heart failure), more than one answer possible.

Question (n)	Response option	Response n (%)
**Actual NYHA class of patients currently using telemonitoring** (n=31)		
	NYHA I	0 (0%)
	NYHA II	19 (61%)
	NYHA III	27 (87%)
	NYHA IV	5 (15%)
**Which NYHA class in your patient population is suitable for applying telemonitoring?** (n=43)		
	NYHA I	3 (6%)
	NYHA II	14 (32%)
	NYHA III	18 (41%)
	NYHA IV	10 (23%)
**Is the NYHA class decisive for applying telemonitoring?** (n=43)		
	Yes	6 (15%)
	No	36 (85%)

In order to determine the best course of therapy, heart failure professionals assess the stage of heart failure according to the New York Heart Association (NYHA) functional classification system (see Table 6). This classification system relates symptoms to everyday activities and the patient’s quality of life. The NYHA class is not a determined factor for the application of telemonitoring according to the guidelines.

**Table 6:** NYHA: New York Heart Association classification for heart failure.

Class	Patient symptoms
Class I (Mild)	No limitation of physical activity. Ordinary physical activity does not cause undue fatigue, palpitation, or dyspnea (shortness of breath).
Class II (Mild)	Slight limitation of physical activity. Comfortable at rest, but ordinary physical activity results in fatigue, palpitation, or dyspnea.
Class III (Moderate)	Marked limitation of physical activity. Comfortable at rest, but less than ordinary activity causes fatigue, palpitation, or dyspnea.
Class IV (Severe)	Unable to carry out any physical activity without discomfort. Symptoms of cardiac insufficiency at rest. If any physical activity is undertaken, discomfort is increased.

Nevertheless, patients in NYHA class II and III were most often reported to be enrolled for telemonitoring, whereas no patients in NYHA class I used telemonitoring. In total, 15% of patients in NYHA class IV used telemonitoring.

#### Length of Time of Telemonitoring

Most respondents stated that they monitor their patients with telemonitoring “as long as needed” or without a time limit. Six clinics noted a maximum time period for using telemonitoring per patient between 3 and 6 months respectively. In response to the question on whether clinics (n=43) could estimate which of the total percentage of all patients in heart failure care were suitable for telemonitoring, the mean percentage was 10%.

#### Telemonitoring System

Fifteen of the 31 clinics that actually used telemonitoring stated that if a new selection process were to be put in place, they would choose a different system compared to the system they currently used. Sixteen clinics indicated that they were not sure which system they would choose (see Table 2). Of the 31 clinics, 14 reported that they were satisfied with their current telemonitoring system. The other 16 clinics took a neutral stance, and one user reported to be dissatisfied with the telemonitoring equipment.

#### Expectations Versus Experienced Outcomes

**Figure 1:**
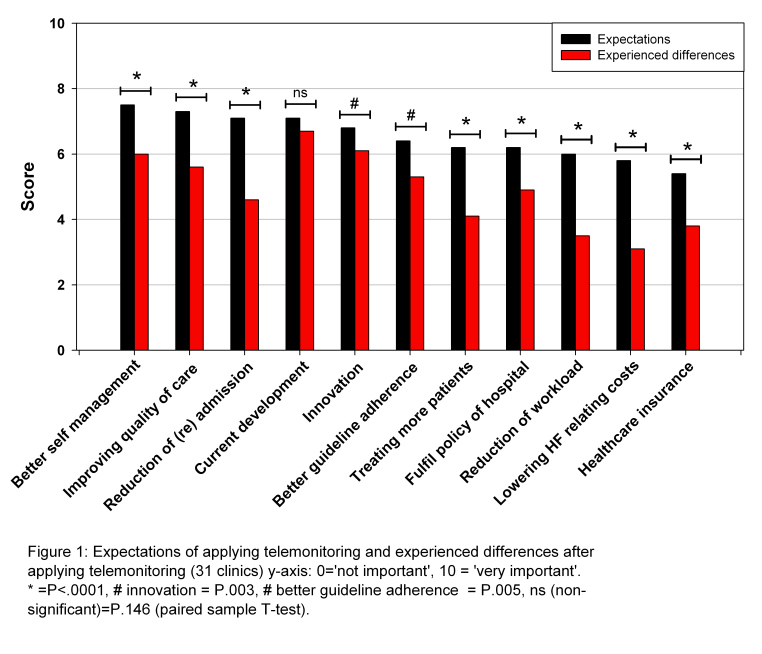
Expectations of applying telemonitoring and experienced differences after implementation of telemonitoring.

In Figure 1, the expectations of applying telemonitoring are compared with the experienced outcomes after implementation of telemonitoring. The combined 3 groups of aspects of working with telemonitoring (direct patient-related care, telemonitoring system aspects, and organizational aspects) and 10 of the 11 separate items showed that the actual experiences did not meet the prior expectations. The results showed that users had high expectations of the benefits of using telemonitoring, in particular with respect to direct patient-care aspects (mean 7.4).

Expectations of the system-related aspects (mean 6.8) and organizational aspects (mean 6.0) were also high. However, these high expectations of the use of telemonitoring were not reflected in the actual experiences after implementation. The largest difference was found in the group of organizational aspects (reduction of workload score, 5.9 versus 3.5, *P*<.001) and lowering heart failure–related costs, score 5.8 versus 3.2, *P*<.001). The aspect “keeping up with current developments” was the only one in which a reduction was not significant (score, 7.2 versus 6.8, *P*=.15).

#### Organizing and Financing Telemonitoring

A total of 12 clinics (39%) reported to be in a “start-up” period; whereas the other 19 clinics stated that they had fully integrated telemonitoring in their daily care routine. Rules and protocols on the implementation of the system and responsibility for incoming data were available in 70% of the clinics. Protocols on the acceptable length of time between the moment of incoming patient data and the response of the caregiver (response-reaction time) were available in 60% of the clinics. With respect to financing, 54% of telemonitoring systems were financed by health care insurance companies, 13% by project financing, and 7% by the hospital itself or the cardiology department. The other 26% of the clinics did not give insight into their financing of telemonitoring.

## Discussion

The most prominent result of our study was that, although the respondents had high perceptions and expectations of working with telemonitoring, these were not positively reflected in the actual experiences.

The trade-offs directly related to the telemonitoring system were most often addressed, but important trade-offs of telemonitoring concerning direct patient care and organizational aspects were only briefly mentioned or not reported at all. A striking finding is that the majority of responding heart failure clinics stated they were considering the use of a different system than the system currently used. Furthermore, aspects of direct patient care (like monitoring and education) were reported as main goals for implementing telemonitoring.

The dominant criteria for using telemonitoring for a specific patient included “education”, “heart failure (re) admission”, and “complaints of heart failure symptoms”. Thirty percent of the respondents mentioned that the actual NYHA class is a criterion for applying telemonitoring, but at the same time only 15% stated that the NYHA class was decisive for applying telemonitoring. In actual practice, the majority of the patients showed to be in NYHA class II and III. Finally, although 1 out of 10 patients was suitable for telemonitoring, the actual number of patients using telemonitoring was limited in general and the duration of the use of telemonitoring unknown. Despite the increased introduction and use of telemonitoring in heart failure, there has been little research regarding user-related aspects of working with telemonitoring. Therefore, it is unknown to what extent expectations, experiences, and possible difficulties in the implementation process of telemonitoring are present in health care providers working with telemonitoring. In this first study to focus specifically on the application of telemonitoring in heart failure clinics, we showed that heart failure clinics have high expectations of patient care, system, and organizational outcomes of working with telemonitoring.

In an earlier study on the expectations of telemonitoring of caregivers in nursing homes, Chang et al [19] reported that respondents expected the benefits of improved efficiency and quality of care, reduction of medical costs, and a reduced workload. However, experiences of telemonitoring were not measured in the study of Chang et al. Although the evidence for the use of telemonitoring in heart failure patients is still growing [5-8], gaps in knowledge about the use of telemonitoring in heart failure remain [3,20,21]. These gaps in knowledge are mainly caused by the absence of data on adequate patient profiling and the overall cost-effectiveness of telemonitoring.

Despite the presence of conflicting evidence on the usefulness of telemonitoring for heart failure and the lack of data regarding the implementation of telemonitoring, the consequences for health care providers, and the logistic processes in daily practice, more than one-third of all heart failure clinics in the Netherlands have implemented this new technology for some of their heart failure patients. This indicates that health care providers have high expectations of working with telemonitoring and are even willing to start working with telemonitoring in the absence of guidelines, protocols, and solid evidence for its usefulness. The use of telemonitoring, however, is still in its infancy, and many clinics are still searching for a way to provide telemonitoring efficiently and effectively. A similar experience was reported with respect to the selection processes for electronic patient records and other technology tools in health care [22-24]. Users were either extremely positive or negative about their system, and this had a “wait-and-see” effect on potential future users. Negative experiences were reflected in the fact that some users were considering looking for a different system than the system currently used. The need for a different system seems to be primarily driven by the practical usage of the system, which falls short of expectations. Our findings indicate that the actual functionalities of the telemonitoring system itself are of great importance to the respondents. Hence, it is questionable if the feeling of overall disappointment is indeed the result of a failing telemonitoring system or is due to a lack of efficient organization around the implementation of telemonitoring systems.

For future success it is very important to create an efficient organization around a system [13]. In the case of telemonitoring, this means that a system should be integrated in a heart failure clinic in which heart failure nurses [11,25] have a coordinating role and have insight in all aspects of patient care (eg, health care professionals involved, situation at home). Within this setting, the heart failure nurse can take appropriate action on the data received from the telemonitoring system [26,27]. Furthermore, additional training is required in which insight and understanding of receiving data, data handling, evaluating expectations, and effect monitoring are vital [28].

Our data showed that in 61% of the heart failure clinics that actually worked with telemonitoring, it was used only in small cohorts with numbers of 10 to 50 patients. Although this concerns only a limited number of patients, it is important to realize that monitoring 50 heart failure patients (next to the treatment of other heart failure patients) might cause a substantial amount of additional work with respect to logistic adjustment, training on using the system, and the development of protocols on data handling, response time, and treatment. We could therefore predict that implementing telemonitoring will not automatically decrease workload.

In this first study on user-related aspects of telemonitoring, we demonstrated that the optimal use of telemonitoring remains a challenge. The main finding of our research is that a substantial difference exists between prior expectations of telemonitoring and the actual use of telemonitoring in daily practice. The focus on, for instance, optimizing medication by using telemonitoring, however, has been shown to be a promising and cost-effective future application [29,30]. While the use of telemonitoring is still in its infancy, it is important to learn from current experiences, even if it currently concerns only a limited number of telemonitoring systems and patients. Ongoing studies such as the IN TOUCH trial [14] in the Netherlands should provide more evidence about cost-effectiveness and the effects of telemonitoring in combination with different types of disease management in heart failure.

A finding that has to be specifically addressed is that most of the respondents indicated that telemonitoring will be applied as long as needed or can even be used indefinitely. This approach should be critically evaluated. First, it might not be the most cost effective in terms of using equipment and staff. Most intervention studies on the use of telemonitoring were short in follow-up, and therefore there are no data available that support the choice for (life) long use of telemonitoring. Second, ethical issues can be raised about whether or not patients would benefit from lifelong monitoring, regardless of the burden on their personal lives. Other notable findings were that 85% of the respondents indicated that the NYHA functional class was not decisive for the application of telemonitoring and that most patients who received telemonitoring were in NYHA functional classes II and III. Although the optimal patient profile for successful use of telemonitoring has not yet been described, it can be expected that specifically patients with severe and more unstable heart failure are suitable for telemonitoring and would benefit in terms of preventing re-admissions. Considering this, it is remarkable that in daily practice telemonitoring is increasingly used for patient education and for optimizing medication in patients with less severe heart failure.

### Limitations

For this study, we used a self-developed questionnaire that was not designed to test the feasibility of a telemonitoring system, but rather to examine both the general considerations and reasons for applying telemonitoring in Dutch heart failure clinics, as well as the organizational aspects these systems address. In this study, we did not focus on possible differences in the perception of working with telemonitoring of heart failure nurses and cardiologists, because the main goal of this study was to explore the expectations and experiences of a heart failure team working with telemonitoring. However, one might predict that the comments of the two separate groups would relate to their characteristics. Although we are aware of the limitations of asking about experiences with telemonitoring retrospectively, the design of this study could not correct for this. To account for this limitation, we have focused in the discussion on the learning aspects of the experiences instead of giving clear-cut conclusions.

### Conclusion

This representative study (86 of 109 surveyed Dutch heart failure clinics) showed that one- third of heart failure clinics were using or planned to use telemonitoring as part of their care, albeit in a limited number of patients only. Our survey also showed that telemonitoring is not a success story yet. Respondents did not experience a decreased workload while working with telemonitoring, and prior expectations of introducing telemonitoring were not reflected in actual experiences, possibly leading to disappointment. Criteria for both the optimal duration period of using the telemonitoring system and the targeted patient groups were not established, and the choice for a telemonitoring system seemed to be made on the specifications of the system itself, rather than on organizational issues such as protocols or education of staff. All the suppliers of telemonitoring devices observed in this study provide the services of generating and transferring data from a home environment to a health care environment. Telemonitoring is not a “one size fits all” solution. From a patient point of view [9,10] and supported by the recent European Society of Cardiology heart failure guidelines (2012), we conclude that the optimal profile of patients who might benefit from telemonitoring needs to be further explored. Long-term experiences are necessary to discover the most effective use of telemonitoring in terms of reduction of mortality, re-admissions, and improvement of quality of life.
